# Japanese nationwide survey to track the impact of long COVID over 3 years

**DOI:** 10.1265/ehpm.25-00293

**Published:** 2025-10-28

**Authors:** Takuya Ozawa, Hideki Terai, Hiromu Tanaka, Arisa Iba, Mariko Hosozawa, Miyuki Hori, Yoko Muto, Eiko Yoshida-Kohno, Ho Namkoong, Shotaro Chubachi, Ryo Takemura, Kengo Nagashima, Yasunori Sato, Makoto Ishii, Hiroyasu Iso, Koichi Fukunaga, the Japan long COVID research group

**Affiliations:** 1 Division of Pulmonary Medicine, Department of Internal Medicine, Keio University School of Medicine, 35 Shinano-machi, Shinjuku-ku, Tokyo 160-8582, Japan; 2 Keio Cancer Center, Keio University School of Medicine, 35 Shinano-machi, Shinjuku-ku, Tokyo 160-8582, Japan; 3 Institute for Global Health Policy Research, Bureau of Global Health Cooperation, Japan Institute for Health Security, 1-21-1 Toyama, Shinjuku-ku, Tokyo 162-8655, Japan; 4 Department of Infectious Diseases, Keio University School of Medicine, 35 Shinano-machi, Shinjuku-ku, Tokyo 160-8582, Japan; 5 Department of Biostatistics, Keio University School of Medicine, 35 Shinano-machi, Shinjuku-ku, Tokyo 160-8582, Japan; 6 Department of Respiratory Medicine, Nagoya University Graduate School of Medicine, 65 Tsurumai-cho, Showa-ku, Nagoya 466-8550, Japan

**Keywords:** Coronavirus disease 2019, COVID-19, Long COVID, Quality of life

## Abstract

**Background:**

The long-term impact of symptom classification on quality of life (QOL) and economic outcomes among individuals with long coronavirus disease (COVID) remains poorly understood. This study aimed to clarify the situation of long COVID in Japan by analyzing patients using cluster classification.

**Methods:**

This multicenter, retrospective cohort study enrolled 515 patients with COVID-19 and followed up for 36 months via standardized questionnaires. Patients were classified based on: 1) symptom trajectory over time and 2) symptom cluster profiles at 3 months.

**Results:**

While the number of symptoms decreased, fatigue and dyspnea frequently persisted, whereas anosmia and dysgeusia declined. Cough and sputum decreased gradually. The proportion of patients with 5–9 symptoms increased. The mean (interquartile range) presenteeism scores were lower in the continuous (60 [50–80]) and relapse groups (65 [48–80]) than in the recovered group (70 [50–80]). The multiple symptoms cluster had the worst SF-36, presenteeism, and absenteeism scores (47.2 [44.7–49.8], 48.8 [27.5–72.5], and 10.9 [0.0–11.0], respectively).

**Conclusions:**

Patients with continuous and multiple symptoms experienced persistently lower QOL and greater economic burden up to 36 months after COVID-19 diagnosis. The long-term effects of long COVID are not only physical but also mental and economical. Thus, further research is needed to clarify the economical and physiological impact of long COVID.

**Supplementary information:**

The online version contains supplementary material available at https://doi.org/10.1265/ehpm.25-00293.

## Background

Even 4 years after the outbreak of the novel coronavirus disease in 2019 (COVID-19), a significant number of patients still experience long-term symptoms after severe acute respiratory syndrome coronavirus 2 (SARS-CoV-2) infection, commonly referred to as “long COVID,” [1] which continues to have socioeconomic impacts. While studies investigating long COVID up to 3 years after the first wave remain limited, existing research highlights several important findings. These include a decrease in the overall frequency of long COVID symptoms over time, with certain symptoms such as fatigue, dyspnea, and alopecia persisting; a higher risk of long COVID symptoms among hospitalized patients than among non-hospitalized patients; and a significant decline in quality of life (QOL) attributed to long COVID symptoms [2, 3]. Even with the Omicron variant, which has a less severe acute phase, it is unclear whether the incidence of long COVID is also reduced. Although research focus has shifted to the current Omicron variants rather than earlier, more virulent strains, it remains crucial to clarify the long-term impact of the initial waves of infection.

Long COVID is still debated, suggesting that it could be a complicated condition to understand [4, 5]. In a systematic review [6, 7], fatigue was reported as the most common symptom, and long COVID affected not only physical health but also mental health, such as anxiety and depression. Several cluster analyses have been conducted to understand this mechanism; specifically, there were two clusters where symptoms recovered or persisted [8, 9]. It is expected that analyzing patients by dividing them into specific clusters, rather than analyzing them as a whole, may lead to a better understanding of long COVID.

Large-scale reports on long COVID over 3 years have been limited. Moreover, the number of studies that have examined the long-term economic impact is also scarce. Thus, this study aimed to clarify the situation of long COVID in Japan by analyzing patients using cluster classification.

## Methods

### Study design and participants

This multicenter, retrospective cohort study included patients from our previous study [10], recruited from 26 medical facilities across Japan (Hokkaido, Tochigi, Saitama, Tokyo, Kanagawa, Aichi, Osaka, and Fukuoka), covering urban, suburban, and rural areas [11]. Briefly, we included patients who were aged ≥18 years, diagnosed with COVID-19 via SARS-CoV-2 polymerase chain reaction or antigen test, and hospitalized during the first to third waves of COVID-19 between January 2020 and February 2021. Patients were asked to complete a questionnaire on paper or via a smartphone application regarding their symptoms associated with long COVID at 36 months after diagnosis. The definition of long COVID at 36 months was the presence of symptoms that persisted after COVID-19 diagnosis or recurred after discharge, in accordance with the definition of Centers for Disease Control and Prevention [12]. The survey period was from November 2023 to February 2024. In addition to the 24 symptoms investigated in a previous study, seven symptoms (nasal discharge, chest pain, palpitations, anorexia, nausea or vomiting, erectile dysfunction, and menstrual changes) were included. Additionally, information on anxiety and depression (assessed by the Hospital Anxiety and Depression Scale), health-related QOL (assessed by the SF-8 scale), economic conditions, and vaccination status were gathered. Economic conditions were assessed using presenteeism and absenteeism scores from the World Health Organization Health and Work Performance Questionnaire. The specific questionnaire items and calculation methods are provided in Supplementary Appendix 1 (Additional file).

To confirm consent to participate in the study, an explanatory letter and a return envelope indicating the willingness to participate and cooperate were sent. This study was approved by the Ethics Committee of Keio University School of Medicine (approval number: 20200243). Each participating institution obtained approval and permission to conduct this study from the institutional ethical review committee or a specified nonprofit organization of the Japan Clinical Research Council.

### Patient enrollment

In the original cohort study, informed consent was obtained from 1,196 participants. We excluded those whose clinical information from each facility was obtained using an electronic data capture system or whose questionnaire results were lacking (n = 130). The number of participants with clinical information and patient-reported outcomes at 3 months was 1,066. Moreover, we excluded patients who were lost to follow-up (n = 39), died (n = 8), or provided reversed informed consent due to reasons such as being too old to answer (n = 43) and others (n = 2). We also excluded the patients whose reported outcomes at 6, 12, or 36 months after diagnosis were lacking (n = 459). Finally, data from 515 patients were included in the analysis. A consort diagram is presented in Fig. S1 (Additional file). Regarding ethnicity, 513 patients were Japanese, and 2 were from other countries. Figure S2 (Additional file) shows the chronological changes in the individual symptoms from 3 to 36 months. The cases were classified into five groups according to the pattern of appearance of long COVID as follows: “Continuous,” symptoms always present; “Recovered,” symptoms disappeared after a certain point during follow-up; “Relapse,” symptoms present at admission, disappeared once, then reappeared; “Late-onset,” no symptoms at admission but symptoms appeared after 3 months; and “Never,” symptoms always absent. Furthermore, based on the cluster analysis of symptoms at 3 months using the same patient group [9], the current cases were classified into clusters. The clusters were named according to the previous cluster analysis, as follows: minimal symptom, anosmia and dysgeusia, fatigue and dyspnea, cough and sputum, and multiple symptoms clusters.

### Statistical analysis

Data were analyzed using JMP Pro 17.2 program (SAS Institute Inc., Cary, NC, USA). Continuous variables are presented as mean and interquartile range (IQR), while categorical variables are presented as the number and percentage of cases. Using the 24 symptom variables evaluated at 3 months post-infection, hierarchical cluster analysis was performed by the Ward’s minimum-variance method, as described in our previous study [9]. The method for calculating absolute presenteeism and absenteeism is presented in the Appendix. Continuous variables were analyzed using Mann–Whitney U (two groups) or Kruskal–Wallis (exceeding two groups) tests, and categorical variables using Pearson’s χ^2^ test. Two-tailed p-values <0.05 indicated statistical significance.

## Results

Table 1 represents the characteristics of patients with long COVID at 36 months after diagnosis compared with those without symptoms. No differences were observed in age, sex, or disease severity between the groups. Regarding comorbidities, the prevalence of asthma was higher in the long COVID group than in the asymptomatic group (n = 13 [10.1%] vs 14 [3.7%], p < 0.01), whereas the prevalence of cardiovascular disease was lower (n = 1 [0.8%] vs 25 [6.6%], p < 0.01), although the number of patients with each comorbidity was small. The proportion of patients admitted to the intensive care unit did not differ between the groups; however, the use of mechanical ventilation was higher (n = 12 [9.6%] vs 15 [4.0%], p = 0.02) in the long COVID group than in the asymptomatic group. Reinfection was observed in 34 patients (27.0%) in the long COVID group and in 87 patients (23.5%) in the asymptomatic group, with no significant difference (p = 0.42). To address the potential impact of dropouts over time, we compared patient characteristics at 3 and 36 months after diagnosis to identify differences between the retained and lost participants (Table S1, Additional file). There were no clear differences between the two groups. Furthermore, symptoms at 36 months were compared between patients with and without reinfection (Table S2, Additional file). Fever was more frequent in the reinfection group (n = 7 [5.8%] vs n = 5 [1.3%], p = 0.006), while the percentage of other symptoms showed no differences.

**Table 1:** Patient characteristics according to the presence or absence of long COVID at 36 months

	**Long COVID at 36 months**		

	**+ (n = 132)**	**− (n = 383)**	**p value**
Age	60 (57–63)	59 (57–60)	0.64
Sex, male	88 (66.7)	230 (60.1)	0.18
Severity			0.35
Asymptomatic	2 (1.6)	7 (1.9)	
Mild	16 (12.8)	72 (19.1)	
Moderate I	55 (44.0)	173 (45.9)	
Moderate II	37 (29.6)	84 (22.3)	
Severe	15 (12.0)	41 (10.9)	
Comorbidities			
Hypertension	48 (36.9)	139 (36.4)	0.91
Diabetes	24 (18.8)	65 (17.2)	0.68
Cardiovascular disease	1 (0.8)	25 (6.6)	0.01
Malignancy	9 (6.9)	22 (5.8)	0.67
Autoimmune disease	3 (2.3)	6 (1.6)	0.58
COPD	6 (4.7)	11 (2.9)	0.33
Asthma	13 (10.1)	14 (3.7)	0.005
Hyperuricemia	16 (12.4)	46 (12.1)	0.92
Chronic liver disease	4 (3.1)	13 (3.4)	0.85
Chronic kidney disease	5 (3.9)	19 (5.0)	0.60
Admission to ICU	15 (12.0)	39 (10.3)	0.60
Mechanical ventilation	12 (9.6)	15 (4.0)	0.016
Past history			
Infection	10 (7.6)	17 (4.4)	0.16
Mental disease	7 (5.3)	10 (2.6)	0.14
Respiratory disease	12 (9.1)	20 (5.2)	0.11
Reinfection	34 (27.0)	87 (23.5)	0.42
Number of symptoms	4.6 (4.3–4.9)	0.0 (−0.2–0.2)	<0.001
Type of symptoms			<0.001
Continuous	79 (59.9)	0 (0)	
Recovered	0 (0)	344 (89.8)	
Relapse	53 (40.2)	39 (10.2)	
Economic condition			<0.001
Become very bad	14 (11.0)	10 (2.7)	
Worse	28 (22.1)	49 (13.0)	
No change	78 (61.4)	295 (78.5)	
Become better	6 (4.7)	15 (4.0)	
Improved very much	1 (0.8)	7 (1.9)	
Absolute presenteeism	58.7 (53.9–63.4)	66.2 (63.5–68.9)	0.003
Absolute absenteeism	−3.7 (−13.0–5.6)	−12.1 (−17.2–6.9)	0.94
SF-36	48.0 (46.9–49.1)	52.1 (51.5–52.8)	<0.001
PCS	46.8 (45.8–47.7)	49.5 (48.8–50.2)	<0.001
MCS	49.1 (44.6–50.0)	52.8 (52.1–53.4)	<0.001

Abbreviations: COPD, chronic obstructive pulmonary disease; ICU, intensive care unit; MCS, mental component survey; PCS, physical component survey; SF-36, MOS 36-item short-form health survey

Missing values were excluded. Continuous variables are shown as the mean (95% confidence interval) and categorical variables as n (%).

**Fig. 1:**
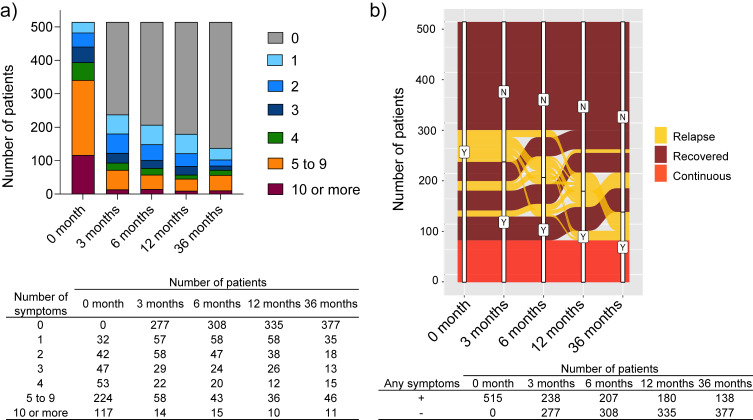
Chronological changes from diagnosis to 36 months after onset of long COVID a) Changes in the number of long COVID cases over time. b) Trends of 515 patients with at least one symptom at each time point. COVID-19, coronavirus disease 2019

Figure 1a illustrates the changes over time in the proportion of patients reporting the indicated number of symptoms, showing the trends across the follow-up period. Compared to the acute phase of infection, the number of patients reporting any symptoms decreased by half at 3 months (n = 238 [46.2%]), then gradually decreased further at 6, 12, and 36 months (n = 207 [40.2%], n = 180 [35.0%], and n = 138 [26.8%], respectively). Thus, the number of patients reporting any symptoms decreased from 3 to 36 months, except for those reporting 5–9 and ≥10 symptoms. Furthermore, a notable increase was identified in the proportion of patients reporting five to nine symptoms between 12 and 36 months (n = 36 [7.0%] and n = 46 [8.9%], respectively). The small number of patients with ≥10 symptoms did not significantly change over time.

Figure 1b shows the trends in patients reporting at least one symptom, categorized into long COVID symptom subgroups: 92 (17.9%), 344 (66.8%), and 79 (15.3%) patients were categorized into the “relapse,” “recovered,” and “continuous,” subgroups, respectively, at 36 months. At 36 months, a substantial proportion of the patients continued to have persistent or relapsed symptoms. Although the prevalence of most symptoms showed a declining trend, certain symptoms, including fatigue, dyspnea, muscular weakness, alopecia, and cough, persisted over time (Fig. S2, Additional file).

The absolute numbers of representative symptoms, such as fatigue, memory impairment, poor concentration, and alopecia, are documented for up to 36 months (Fig. S3, Additional file). In addition, the corresponding percentages for each symptom, as well as the percentage of patients with at least one symptom, are also shown (Fig. S4, Additional file). Patients with no symptoms at any time point were excluded for clear interpretation. Symptoms remained continuous in a certain number of patients, and patients with relapsed or late-onset symptoms were observed as late as 36 months.

We classified the five clusters based on the results of the cluster analysis of symptoms at 3 months using the same patient group. Patients were classified into the same clusters defined by their symptoms at 3 months after diagnosis (Table 2). The mean (IQR) number of symptoms was the smallest in the minimal symptoms cluster and the largest in the multiple symptoms cluster at 0.5 (0.2–0.8) and 5.0 (4.0–6.0), respectively. Similarly, the mean (IQR) SF-36 QOL score was the highest in the minimal symptoms cluster and the lowest in the multiple symptoms cluster at 52.2 (51.4–52.9) and 47.2 (44.7–49.8), respectively. The absolute presenteeism score was higher in the multiple symptoms cluster than in the other clusters. The multiple symptoms cluster mostly comprised the continuous group, whereas the other clusters mainly comprised the recovered group. In the multiple symptom cluster, the symptoms tended to persist for 36 months.

**Table 2:** Patient characteristics compared by cluster

	**Cluster**					

	**Minimal** **symptoms**	**Anosmia and** **Dysgeusia**	**Fatigue and** **Dyspnea**	**Cough and** **Sputum**	**Multiple** **symptoms**	**p value**
	**n = 307**	**n = 45**	**n = 111**	**n = 26**	**n = 26**	
Age	61 (59–63)	55 (51–59)	55 (52–57)	62 (59–67)	60 (55–66)	<0.001
Sex, male	199 (64.8)	21 (46.7)	66 (59.5)	15 (57.7)	17 (65.4)	0.19
Severity						0.017
Asymptomatic	6 (2.0)	1 (2.3)	1 (0.9)	1 (3.9)	0 (0)	
Mild	48 (16.0)	17 (38.6)	19 (17.9)	2 (7.7)	2 (7.7)	
Moderate I	146 (48.7)	17 (38.6)	44 (41.5)	12 (46.2)	9 (34.6)	
Moderate II	70 (23.3)	7 (15.9)	30 (28.3)	6 (23.1)	8 (30.8)	
Severe	30 (10.0)	2 (4.6)	12 (11.3)	5 (19.2)	7 (26.9)	
Comorbidities						
Hypertension	124 (40.5)	9 (20.0)	35 (32.1)	11 (42.3)	8 (30.8)	0.059
Diabetes	60 (19.7)	1 (2.3)	18 (16.7)	5 (19.2)	5 (19.2)	0.09
Cardiovascular disease	18 (5.9)	4 (8.9)	2 (1.9)	1 (3.9)	1 (3.9)	0.37
Malignancy	23 (7.6)	2 (4.4)	3 (2.8)	2 (7.7)	1 (3.9)	0.43
Autoimmune disease	2 (0.7)	2 (4.4)	5 (4.6)	0 (0)	0 (0)	0.04
COPD	9 (3.0)	0 (0)	3 (2.8)	2 (7.7)	3 (11.5)	0.069
Asthma	15 (4.9)	4 (9.1)	4 (3.7)	1 (3.9)	3 (11.5)	0.40
Hyperuricemia	39 (12.8)	5 (11.4)	13 (12.0)	2 (7.7)	3 (11.5)	0.96
Chronic liver disease	7 (2.3)	2 (4.4)	6 (5.6)	1 (3.9)	1 (3.9)	0.58
Chronic kidney disease	16 (5.3)	3 (6.7)	5 (4.6)	0 (0)	0 (0)	0.53
Admission to ICU	31 (10.3)	2 (4.6)	11 (10.4)	5 (19.2)	5 (20.0)	0.20
Mechanical ventilation	9 (3.0)	1 (2.3)	9 (8.5)	3 (11.5)	5 (20.0)	<0.001
Past medical history						
Infection	9 (2.9)	1 (2.2)	13 (11.7)	2 (7.7)	2 (7.7)	0.007
Mental disease	6 (2.0)	1 (2.2)	8 (7.2)	1 (3.9)	1 (3.9)	0.12
Respiratory disease	16 (5.2)	3 (6.7)	5 (4.5)	4 (15.4)	4 (15.4)	0.072
Number of symptoms	0.5 (0.2–0.8)	1.2 (0.4–1.9)	1.9 (1.4–2.3)	2.5 (1.5–3.5)	5.0 (4.0–6.0)	<0.001
Presence of symptoms	42 (13.7)	15 (33.3)	47 (42.3)	11 (42.2)	17 (65.4)	<0.001
Type of symptoms						<0.001
Continuous	10 (3.3)	11 (24.4)	28 (25.2)	10 (38.5)	15 (57.7)	
Recovered	232 (75.6)	30 (66.7)	63 (56.8)	14 (53.9)	10 (38.5)	
Relapse	65 (21.2)	4 (8.9)	20 (18.0)	2 (7.7)	1 (3.9)	
Economic condition						<0.001
Become very bad	9 (2.9)	1 (2.2)	7 (6.3)	3 (11.5)	4 (15.4)	
Worse	27 (8.8)	6 (13.3)	29 (26.1)	7 (26.9)	8 (30.8)	
No change	247 (80.5)	33 (73.3)	64 (57.7)	16 (61.5)	13 (50.0)	
Become better	12 (3.9)	3 (6.7)	5 (4.5)	0 (0)	1 (3.9)	
Improved very much	4 (1.3)	0 (0)	4 (3.6)	0 (0)	0 (0)	
Absolute presenteeism	66.6 (50.0–80.0)	68.8 (60.0–80.0)	62.1 (50.0–80.0)	57.5 (50.0–70.0)	48.8 (27.5–72.5)	0.011
Absolute absenteeism	−14.8 (−20.0–0.0)	−0.9 (0.0–0.0)	−6.7 (−20.0–0.0)	−11.4 (−20.0–0.0)	10.9 (0.0–11.0)	0.008
SF-36	52.2 (51.4–52.9)	50.9 (48.9–52.8)	49.1 (47.8–50.3)	51.2 (48.6–53.8)	47.2 (44.7–49.8)	<0.001
PCS	49.2 (48.5–49.9)	48.4 (46.5–50.3)	47.7 (46.5–48.9)	48.6 (46.1–51.1)	44.5 (42.0–47.0)	0.001
MCS	52.8 (52.1–53.5)	51.6 (49.8–53.3)	49.1 (48.0–50.3)	50.0 (47.7–52.3)	47.8 (45.5–50.2)	<0.001

Abbreviations: COPD, chronic obstructive pulmonary disease; ICU, intensive care unit; MCS, mental component survey; PCS, physical component survey; SF-36, MOS 36-item short-form health survey

Missing values were excluded. Continuous variables are shown as the mean (95% confidence interval) and categorical variables as n (%).

**Fig. 2:**
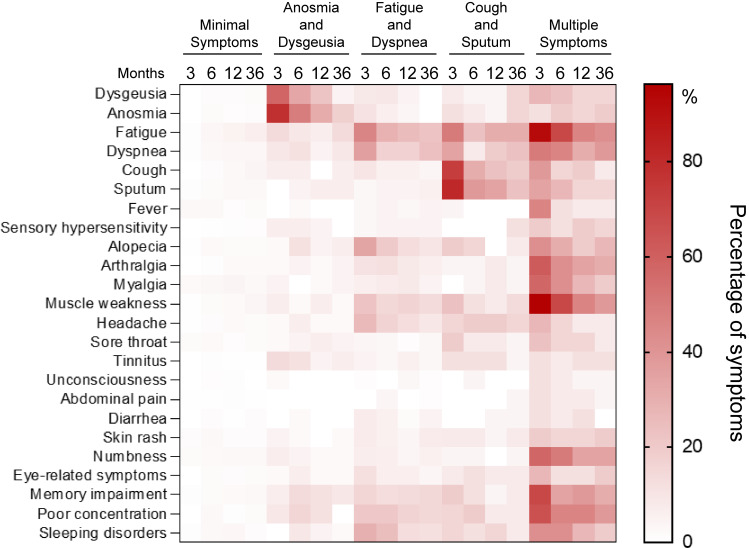
Heatmap of the frequency of long COVID cases compared by clusters COVID-19, coronavirus disease 2019

Figure 2 shows the trend in the frequency of each long COVID symptom by cluster as a heatmap. In the minimal symptom cluster, symptom frequencies remained consistently low across all time points from 3 to 36 months, with no notable changes during the follow-up period. In the anosmia and dysgeusia cluster, the frequencies of anosmia and dysgeusia remained elevated at 12 months (n = 14 [41.1%] and 10 [22.2%]) but declined at 36 months (n = 8 [17.8%] and 2 [4.4%]). In the fatigue and dyspnea group, fatigue and dyspnea were relatively frequent at 12 months (n = 28 [25.2%] and 19 [17.1%]). At 36 months, fatigue decreased (n = 25 [22.5%]), but dyspnea increased (n = 26 [23.4%]). In the cough and sputum cluster, both symptoms were more common at 12 months (n = 6 [23.1%] and 9 [34.6%]), and decreased slightly at 36 months (n = 5 [19.2%] and 6 [23.1%]). In contrast, the number of patients in the multiple symptoms cluster remained high for many symptoms, such as fatigue (n = 11 [42.3%]), dyspnea (n = 10 [38.5%]), alopecia (n = 7 [26.9%]), arthralgia (n = 8 [30.8%]), muscle weakness (n = 10 [38.5%]), numbness (n = 9 [34.6%]), eye-related symptoms (n = 5 [19.2%]), memory impairment (n = 8 [30.8%]), poor concentration (n = 10 [38.5%]), and sleep disorder (n = 5 [19.2%]).

In the longitudinal analysis of patients with at least one symptom (Fig. 1a), the proportion of patients with five to nine symptoms increased between 12 and 36 months. Accordingly, we compared patients with five or more symptoms with those with less than five symptoms. Owing to our focus on patients who had any symptoms, we excluded those who had zero symptoms (n = 377) from the analysis and compared groups of patients with one to four symptoms (n = 81) and five or more symptoms (n = 57).

**Fig. 3:**
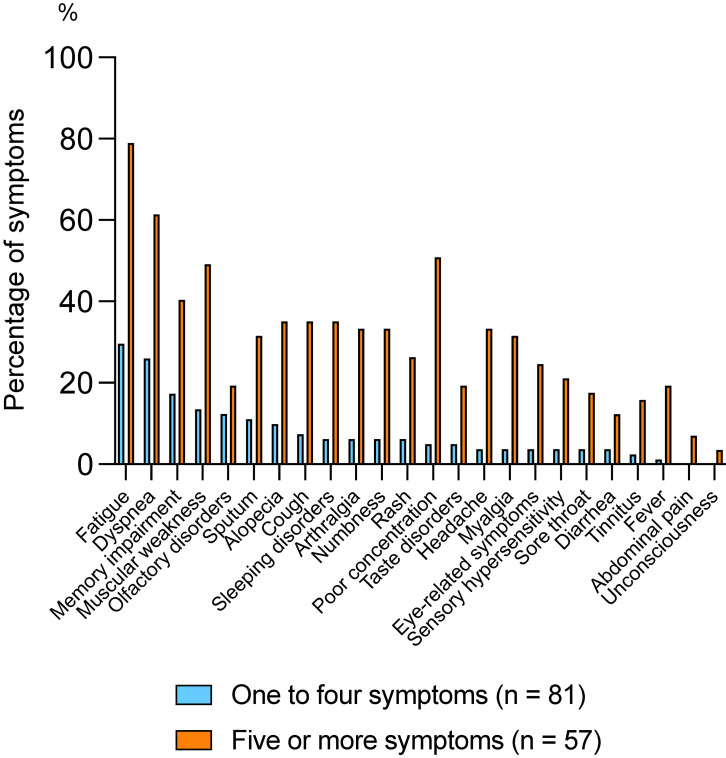
Comparison of the proportion of each symptom in patient groups stratified by number of symptoms

Figure 3 shows the comparison between these groups. In all symptoms, the proportion of patients with the symptom was significantly higher in patients with five or more symptoms than those with one to four symptoms. Fatigue, dyspnea, memory impairment, and muscle weakness were the most common symptoms in both groups. Patients with five or more symptoms were more likely to have symptoms such as poor concentration, alopecia, cough, and sleep disorders than those with one to four symptoms.

The patient characteristics, comparing those with five or more symptoms, to those with one to five symptoms, are summarized (Table S3, Additional file). In patients with five or more symptoms, compared with those with fewer symptoms, the proportion whose economic conditions “become very bad” or “become worse” was higher: 47.3% (n = 26) vs 24.3% (n = 19); the mean absenteeism score was higher: 3.5 (IQR: 13.1–20.0) vs −9.8 (IQR: −21.4–1.8); and the mean SF-36 QOL score was lower: 45.3 (IQR: 43.2–47.5) vs 50.3 (IQR: 48.6–52.0).

**Fig. 4:**
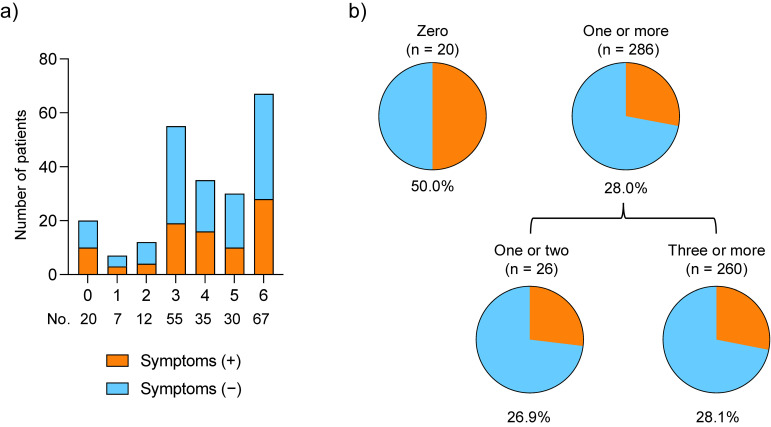
Comparison of vaccination frequency and symptoms among symptomatic patients at 3 months a) Number of vaccinations and symptoms. b) Proportion of long COVID cases by vaccination status. COVID-19, coronavirus disease 2019

Figure 4 shows the number of vaccinations received and proportion of long COVID cases in symptomatic patients at 3 months. When comparing the number of vaccinations received, long COVID was less common in patients who received at least one vaccination (n = 80 [28.0%]) than in those who received no vaccinations (n = 10 [50.0%]). However, no significant difference in the prevalence of long COVID was observed between patients who received fewer than three vaccinations and those who received three or more vaccinations: 26.9% (n = 7) and 28.1% (n = 73), respectively.

**Fig. 5:**
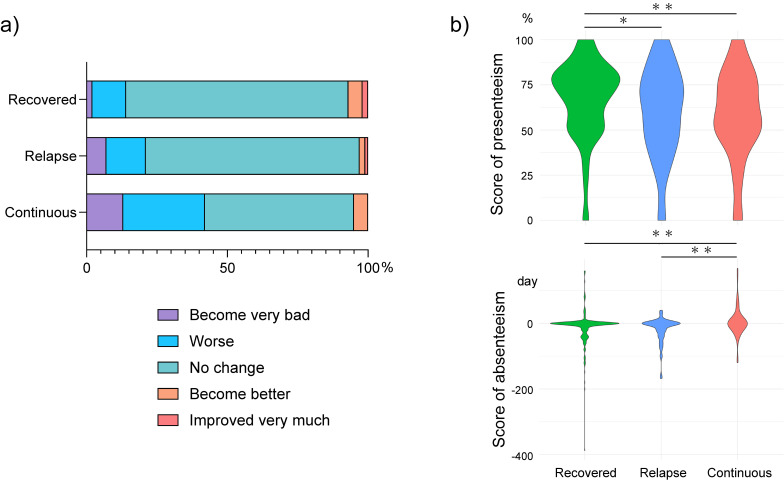
Comparison of economic conditions by type of long COVID symptom transition a) Change in subjective economic condition. b) The upper row is absolute presenteeism and lower row indicates absolute absenteeism. COVID-19, coronavirus disease 2019

Figure 5a shows the changes in subjective economic conditions according to the patterns of symptom presentation during long COVID. The proportion of patients who answered “become very bad” and “become worse” was the highest in the continuous group followed by the relapse and recovered groups at 43.6% (n = 31), 21.1% (n = 19), and 14.9% (n = 51), respectively. The proportion who answered “no change” was almost the same for the relapse and recovered groups at 75.6% (n = 68) and 78.6% (n = 265), respectively, whereas it was significantly lower for the continuous group at 52.6% (n = 40; p < 0.001).

Figure 5b compares absolute presenteeism with absenteeism. For absolute presenteeism, both the continuous and relapse groups had lower mean scores compared with the recovered group at 60 (IQR: 50–80), 65 (IQR: 48–80), and 70 (IQR: 50–80) (p < 0.01, p = 0.017), respectively. The mean absolute absenteeism was 0 in all groups, with interquartile ranges (IQRs) of 0–0 for the continuous group, −12–0 for the relapse group, and −20–0 for the recovered group (both p < 0.01).

## Discussion

In this observational study of 515 patients with COVID-19 followed for approximately 3 years after diagnosis, we found that long COVID persisted in a certain number of patients, and patients with multiple symptoms at 3 months (multiple symptoms cluster) also presented them at 36 months. Moreover, this study suggested that long COVID affected not only physical health but also QOL and economic conditions at 36 months; the severity of which varies according to the pattern and course of long COVID. To the best of our knowledge, this is the first study to classify and compare long COVID by symptom clusters and analyze subjective economic conditions, QOL, and work productivity scores.

In our previous analysis conducted for up to 12 months after diagnosis, the risk of long COVID was higher in females than in males, with an odds ratio of 2.54 (95% confidence interval: 1.43–4.53) [10]. However, at 36 months, no sex difference was observed in the proportion of patients with and without long COVID, suggesting that the impact of sex on long COVID decreased after 3 years.

A search of the PubMed database using the keywords “long COVID or PASC” and “three years” in the title or abstract up to December 2024 identified six studies that monitored patients for 36 months after COVID diagnosis (Table S4, Additional file). The results from long-term follow-up studies conducted in China have been reported [2, 13]. Han et al. reported that 728 of 1,359 (54%) patients hospitalized at a Wuhan hospital in China between January and May 2020 had at least one long COVID symptom at the 36-month follow-up [13]. Zhang et al. reported that for patients with residual lung lesions at discharge from Wuhan hospital, 160 of 728 (22%) had persistent respiratory symptoms at the 36-month follow-up [2]. The prevalence of long COVID at 36 months in the present study was lower than that in the previous Chinese studies. The proportion of severe patients who required oxygen supplementation in China was 74.3% (n = 1,004) [2], relative to 34.4% (n = 177) in the present study. Therefore, our sample of Japanese patients may have had less severe infection compared with the Chinese patients.

Regarding the number of symptoms, the proportion of patients with one to four symptoms decreased over time. However, the proportion of patients with 5–9 symptoms increased, and the proportion with ≥10 symptoms remained stable from 12 to 36 months, albeit the number of cases was small. The patients with five or more symptoms may overlap with those in the multiple symptoms cluster categorized in our previous cluster analysis [9]. With respect to psychiatric symptoms, Taquet et al. reported in the UK that patients in the recovered cluster at 6 months experienced few symptoms at 2–3 years post-infection, whereas those in the severe-case cluster exhibited decadent depression, fatigue, and subjective cognitive impairment [14].

Common and persistent symptoms include fatigue, dyspnea, poor concentration, and memory impairment, whereas olfactory and taste disorders, which are often observed in patients with mild diseases [15, 16], diminished over time. Yang et al. reported that 39.8% of patients had at least one symptom at 3 years, and the most common symptoms were fatigue, sleep disorder, joint pain, dyspnea, and muscle pain [17], consistent with the findings of our study. Furthermore, analysis at 36 months using cluster classification in this study suggested that anosmia and dysgeusia tended to decrease, while fatigue, dyspnea, cough, and sputum tended to persist, and each cluster had their characteristics. The mechanisms underlying anosmia and dysgeusia in long COVID remain unclear. One hypothesis suggests that SARS-CoV-2 infection of olfactory mucosal supporting cells or pericyte cells may trigger local inflammation and cytokine release, or that local inflammation caused by SARS-CoV-2 infection of ACE2-expressing cells in the tongue epithelium or salivary glands may be involved [18]. Interestingly, while local symptoms such as anosmia and dysgeusia decreased at 36 months, systemic symptoms such as fatigue and dyspnea tended to persist. This finding indicates that symptoms involving local epithelial symptoms and systemic inflammatory responses in the long-term course of long COVID may have different pathophysiological mechanisms. In fact, regarding the mechanisms underlying fatigue and dyspnea, hypotheses exist suggesting that hyper-inflammatory response associated with SARS-CoV-2 infection trigger abnormal mast cell reactions, or that residual lung injury contributes to these symptoms [19]. Further research is needed to elucidate the pathology.

In the current study, patients with asthma tended to have a long COVID at 36 months, which is consistent with the finding of a previous systematic review and meta-analysis [20] reporting an odds ratio of 1.55 (95% confidence interval, 1.30–1.86) for long COVID among hospitalized patients with asthma. These findings suggest that asthma may increase the risk of long COVID.

Herein, we investigated the association between long COVID and vaccination status because the impact of vaccination on changes in long COVID was limited in the previous study [21]. We found that patients who received one or two vaccinations as well as those who received three or more vaccinations tended to exhibit fewer long COVID symptoms compared with unvaccinated patients. Our result is consistent with the finding of a systematic review suggesting that COVID-19 vaccination may reduce the incidence of long COVID [22].

The finding of poor economic situations at 36 months among patients with five or more symptoms was in line with that from a previous study in 2024, wherein 13.9% of patients had not returned to work at 3 months after the COVID diagnosis; however, when limited to patients with five or more symptoms, 27.8% had not returned to work [1]. Regarding the decrease in presenteeism in the continuous and relapsed groups in the present study, previous studies have reported that presenteeism can decrease by remote working [23] and that productivity can decrease by the interruption of treatment for underlying diseases [24]. Patients who were hospitalized and severely ill were less likely to return to work, had poor SF-36 scores, and could not resume their occupational activities as before [1]. Furthermore, we found a decrease in income among patients with long COVID, implying that long COVID may have a wide-ranging impact on socioeconomic factors.

Herein, presenteeism decreased in both the continuous and relapsed groups; however, absenteeism tended to worsen only in the continuous group. This suggests that when symptoms persist, going to work becomes challenging, and work performance declines. In contrast, when the symptoms do not persist but recur, going to work is easy, but work performance decreases. Notably, presenteeism and absenteeism were superior in the recovered group than in the other groups.

Few large-scale studies have evaluated the impact of COVID-19 over approximately 3 years, and even fewer have investigated its socioeconomic impact. We demonstrated that the patients’ economic condition worsened, particularly for those with many symptoms. Furthermore, no other studies have evaluated how the condition of patients classified by previous cluster analysis has changed over 3 years. We found that patients in the multiple symptoms cluster at 3 months after diagnosis also had multiple symptoms at 36 months post-infection, whereas patients in other clusters lost their characteristic symptoms.

Despite its strengths, this study had certain limitations. First, at 36 months post-infection, some patients may have found it difficult to determine whether their current symptoms were related to COVID-19. Additionally, no clinical investigations such as blood tests or imaging studies were conducted, resulting in a lack of objective indicators. This limitation makes it challenging to differentiate long COVID from SARS-CoV-2 reinfection, particularly in the absence of virological confirmation. Consequently, some cases of symptom recurrence due to reinfection may have been misclassified as long COVID. A certain number of patients were diagnosed with reinfection within 36 months, and fever was more frequent in these patients. Although the questionnaire asked about symptoms since the initial hospitalization, it is possible that the fever reported at 36 months was influenced by recent reinfection. Therefore, future studies should incorporate objective clinical indicators to improve diagnostic accuracy and interpretation of long-term symptoms. Second, healthy controls were not included. Thus, we could not compare the changes occurring in the outcome measures over time between patients and controls to determine any changes unrelated to long COVID. It was unclear whether the changes in economic conditions were due to long COVID or to daily life during the pandemic *per se*. Third, only patients from the first and third waves of the COVID-19 pandemic between January 2020 and February 2021 were included in this study. Therefore, our results cannot be applied to patients with long COVID that developed after that period.

## Conclusions

We clarified the actual long-term situation of long COVID in Japan. Several patients presented with persistent or relapsed symptoms. The long-term effects of long COVID are not only physical but also mental and economical. Thus, further research is needed to clarify the economical and physiological impact of long COVID.

## Abbreviations

COVID-19: coronavirus disease 2019
IQR: interquartile range
QOL: quality of life
SARS-CoV-2: severe acute respiratory syndrome coronavirus 2

## Supplementary information

**Additional file 1:** **Supplementary Appendix 1:** Questionnaire on Economic Conditions. **Table S1.** Patient characteristics according to the presence or absence of long COVID at 36 months. **Table S2.** Symptoms at 36 months compared by reinfection. **Table S3.** Comparison of characteristics between patients with one to four symptoms and those with five or more symptoms. **Table S4.** Other studies researching long COVID up to 36 months. **Figure S1.** Consort diagram. **Figure S2.** Changes in all symptoms determined through questionnaire interview. **Figure S3.** Trends of patients. **Figure S4.** Percentage of patients by pattern of long COVID symptom appearance at each time point.

**Additional file 2:** **Supplementary Appendix 2:** Collaborators.

## Declarations

### Availability of data and material

The datasets generated and analyzed during the current study are not publicly available due to ethical restrictions but are available from the corresponding author upon reasonable request.

### Collaborators

All members of the Japan long COVID research group are listed in Supplementary Appendix 2.
