# Supplementary material for: Japanese nationwide survey to track the impact of long COVID over 3 years
Source: Environ Health Prev Med. 2025 Oct 28;30:84. doi: 10.1265/ehpm.25-00293 (PMC12583970; doi:10.1265/ehpm.25-00293)
Supplement: Supplementary file 1 — Additional file 1: Supplementary Appendix 1: Questionnaire on Economic Conditions. Table S1. Patient characteristics according to the presence or absence of long COVID at 36 months. Table S2. Symptoms at 36 months compared by reinfection. Table S3. Comparison of characteristics between patients with one to four symptoms and those with five or more symptoms. Table S4. Other studies researching long COVID up to 36 months. Figure S1. Consort diagram. Figure S2. Changes in all symptoms determined through questionnaire interview. Figure S3. Trends of patients. Figure S4. Percentage of patients by pattern of long COVID symptom appearance at each time point. [file ehpm-30-084-s001.docx]

**Additional file**

**Supplementary Appendix 1: Questionnaire on Economic Conditions**

Details of the questionnaire on economic conditions are shown below.

Q1: Has your financial situation changed since the first wave of novel coronavirus infectious disease emerged in 2019 (COVID-19)?

Answers were selected as follows: ‘Become very bad,’ ‘Worse,’ ‘No change,’ ‘Become better,’ and ‘Improved very much.’

Q2: How would you rate your overall performance on the days you worked in the past four weeks (28 days)? Please enter the number that best describes your performance, from 0 (worst performance) to 10 (best performance).

Q3: How many hours a week does your employer expect you to work? (If it varies, please estimate the average.)

Q4: How many hours did you work in the past seven days? (If it was 97 hours or more, please write 97.)

Absolute presenteeism was calculated by the answer of Q2 $\times10$.

Absolute absenteeism was calculated by the answers of Q3 $-$Q4.

**Supplementary Appendix 2: Collaborators**

The members of the Japan Long COVID Research Group are listed in a separate file.

**Table S1. Patient characteristics according to the presence or absence of long COVID at 36 months**

|  | At 36 months | At three months |  |
| --- | --- | --- | --- |
|  | n = 515 | n = 1,066 |  |
| Age | 59 (51-70) | 57 (45-70) |  |
| Sex, male | 318 (61.7) | 679 (63.7) |  |
| Severity |  |  |  |
| Asymptomatic | 9 (1.8) | 41 (3.9) |  |
| Mild | 88 (17.5) | 222 (21.3) |  |
| Moderate I | 228 (45.4) | 434 (41.7) |  |
| Moderate II | 121 (24.1) | 237 (22.8) |  |
| Severe | 56 (11.1) | 106 (10.2) |  |
| Comorbidities |  |  |  |
| Hypertension | 187 (36.5) | 344 (32.5) |  |
| Diabetes | 89 (17.6) | 178 (16.9) |  |
| Cardiovascular disease | 26 (5.1) | 68 (6.4) |  |
| Malignancy | 31 (6.1) | 71 (6.7) |  |
| Autoimmune disease | 9 (1.8) | 19 (1.8) |  |
| COPD | 17 (3.3) | 32 (3.0) |  |
| Asthma | 27 (5.3) | 55 (5.2) |  |
| Hyperuricemia | 62 (12.2) | 108 (10.2) |  |
| Chronic liver disease | 17 (3.3) | 35 (3.3) |  |
| Chronic kidney disease | 24 (4.7) | 46 (4.4) |  |
| Admission to ICU | 54 (10.8) | 104 (9.9) |  |
| Mechanical ventilation | 27 (5.4) | 47 (4.5) |  |

Missing values were excluded. Continuous variables are shown as the mean (95% confidence interval) and categorical variables as n (%).

Abbreviations: COPD, chronic obstructive pulmonary disease; ICU, intensive care unit, SF-36, MOS 36-item short-form health survey; PCS, physical component survey; MCS, mental component survey

**Table S2. Symptoms at 36 months compared by reinfection**

|  | Reinfection | |  |
| --- | --- | --- | --- |
| Symptoms | + (n = 121) | － (n = 376) | p value |
| Fever | 7 (5.8) | 5 (1.3) | 0.006 |
| Cough | 7 (5.8) | 17 (4.5) | 0.32 |
| Sputum | 6 (5.0) | 19 (5.1) | 0.97 |
| Dyspnea | 17 (14.1) | 37 (9.8) | 0.20 |
| Sensory disorder | 5 (4.1) | 10 (2.7) | 0.68 |
| Fatigue | 17 (14.1) | 46 (12.2) | 0.27 |
| Alopecia | 5 (4.1) | 22 (5.9) | 0.53 |
| Muscler weakness | 5 (4.1) | 19 (5.1) | 0.17 |
| Myalgia | 7 (5.8) | 14 (3.7) | 0.96 |
| Sensory hypersensitivity | 12 (9.9) | 25 (6.7) | 0.23 |
| Headache | 5 (4.1) | 16 (4.3) | 0.95 |
| Sore throat | 5 (4.1) | 6 (1.6) | 0.099 |
| Tinnitus | 4 (3.3) | 7 (1.9) | 0.35 |
| Unconsciousness | 1 (0.8) | 1 (0.3) | 0.40 |
| Abdominal pain | 0 (0) | 3 (0.8) | 0.32 |
| Diarrhea | 2 (1.7) | 8 (2.1) | 0.75 |
| Rash | 3 (2.5) | 16 (4.3) | 0.38 |
| Numbness | 8 (6.6) | 15 (4.0) | 0.23 |
| Eye-related symptoms | 4 (3.3) | 12 (3.2) | 0.95 |
| Memory impairment | 11 (9.1) | 25 (6.7) | 0.37 |
| Poor concentration | 8 (6.6) | 24 (6.4) | 0.93 |
| Sleeping disorders | 8 (6.6) | 16 (4.3) | 0.29 |
| Dysgeusia | 1 (0.8) | 13 (3.5) | 0.13 |
| Anosmia | 3 (2.5) | 17 (4.5) | 0.32 |
| Missing values were excluded. categorical variables are shown as n (%). | | | |

**Table S3. Comparison of characteristics between patients with one to four symptoms and those with five or more symptoms**

|  | Long COVID at 36 months | |  |
| --- | --- | --- | --- |
|  | One to four symptoms (n = 81) | Five or more symptoms (n = 57) | p value |
| Age | 61 (58–64) | 59 (55–63) | 0.49 |
| Sex, male | 58 (71.6) | 36 (63.2) | 0.29 |
| Severity |  |  | 0.081 |
| Asymptomatic | 0 (0) | 2 (3.7) |  |
| Mild | 12 (15.6) | 4 (7.4) |  |
| Moderate I | 31 (40.3) | 26 (48.2) |  |
| Moderate II | 20 (26.0) | 18 (33.3) |  |
| Severe | 14 (18.2) | 4 (7.4) |  |
| Comorbidities |  |  |  |
| Hypertension | 30 (38.0) | 20 (35.1) | 0.73 |
| Diabetes | 16 (20.5) | 8 (14.3) | 0.35 |
| Cardiovascular disease | 1 (1.3) | 0 (0) | 0.39 |
| Malignancy | 8 (10.1) | 1 (1.8) | 0.053 |
| Autoimmune disease | 2 (2.6) | 1 (1.8) | 0.75 |
| COPD | 4 (5.1) | 2 (3.5) | 0.65 |
| Asthma | 6 (7.7) | 7 (12.3) | 0.37 |
| Hyperuricemia | 11 (14.1) | 5 (8.8) | 0.34 |
| Chronic liver disease | 2 (2.5) | 2 (3.5) | 0.74 |
| Chronic kidney disease | 3 (3.9) | 2 (3.5) | 0.91 |
| Admission to ICU | 12 (15.6) | 5 (9.3) | 0.29 |
| Mechanical ventilation | 13 (16.9) | 2 (3.7) | 0.02 |
| Past history |  |  |  |
| Infection | 6 (7.4) | 5 (8.8) | 0.77 |
| Mental disease | 3 (3.7) | 1 (3.6) | 0.38 |
| Respiratory disease | 6 (7.4) | 7 (12.3) | 0.33 |
| Number of symptoms | 2.1 (1.6–2.6) | 7.7 (6.9–8.5) | <0.001 |
| Type of symptoms |  |  | 0.029 |
| Continuous | 40 (49.4) | 39 (68.4) |  |
| Recovered | 5 (6.2) | 0 (0) |  |
| Relapse | 36 (44.4) | 18 (31.6) |  |
| Economic condition |  |  | 0.027 |
| Become very bad | 4 (5.1) | 10 (18.2) |  |
| Worse | 15 (19.2) | 16 (29.1) |  |
| No change | 55 (70.5) | 26 (47.3) |  |
| Become better | 4 (5.1) | 2 (3.6) |  |
| Improved very much | 0 (0) | 1 (1.8) |  |
| Absolute presenteeism | 63.5 (57.1–69.9) | 53.3 (45.1–61.5) | 0.14 |
| Absolute absenteeism | -9.8 (-21.4–1.8) | 3.5 (-13.1–20.0) | 0.036 |
| SF-36 | 50.3 (48.6–52.0) | 45.3 (43.2–47.5) | <0.001 |
| PCS | 47.6 (46.2–49.1) | 43.4 (41.5–45.3) | <0.001 |
| MCS | 51.6 (50.2–53.1) | 46.8 (44.9–48.7) | <0.001 |

Missing values were excluded. Continuous variables are shown as the mean (95% confidence interval) and categorical variables as n (%).

Abbreviations: COPD, chronic obstructive pulmonary disease; ICU, intensive care unit, SF-36, MOS 36-item short-form health survey; PCS, physical component survey; MCS, mental component survey

**Table S4. Other studies researching long COVID up to 36 months**

|  | Author | Journal |  |  |
| --- | --- | --- | --- | --- |
| #1 | Cai M, et al. | Nat Med 2024;30:1564-1573. | | |
| #2 | Zhang H, et al. | Lancet Respir Med 2024;12:55-66. | | |
| #3 | Taquet M, et al. | Lancet Psychiatry 2024;11:696-708. | | |
| #4 | Yang H, et al. | J Med Virol 2024;96:e29566. | | |
| #5 | Eligulashvili A, et al. | PLoS Med 2024;21:e1004263. | | |
| #6 | Han X, et al. | Eur Respir J 2024;22:2301612. | | |

**Figure S1. Consort diagram.**

**Figure S2. Changes in all symptoms determined through questionnaire interview**

**Figure S3. Trends of patients**

Trends of patients with a) fatigue, b) memory impairment, c) concentration impairment, and d) alopecia at each time point, including no symptoms at all time points. Y is the number of patients with symptoms, and N is the number of patients without symptoms. Patients with no symptoms were excluded.

**Figure S4. Percentage of patients by pattern of long COVID symptom appearance at each time point**


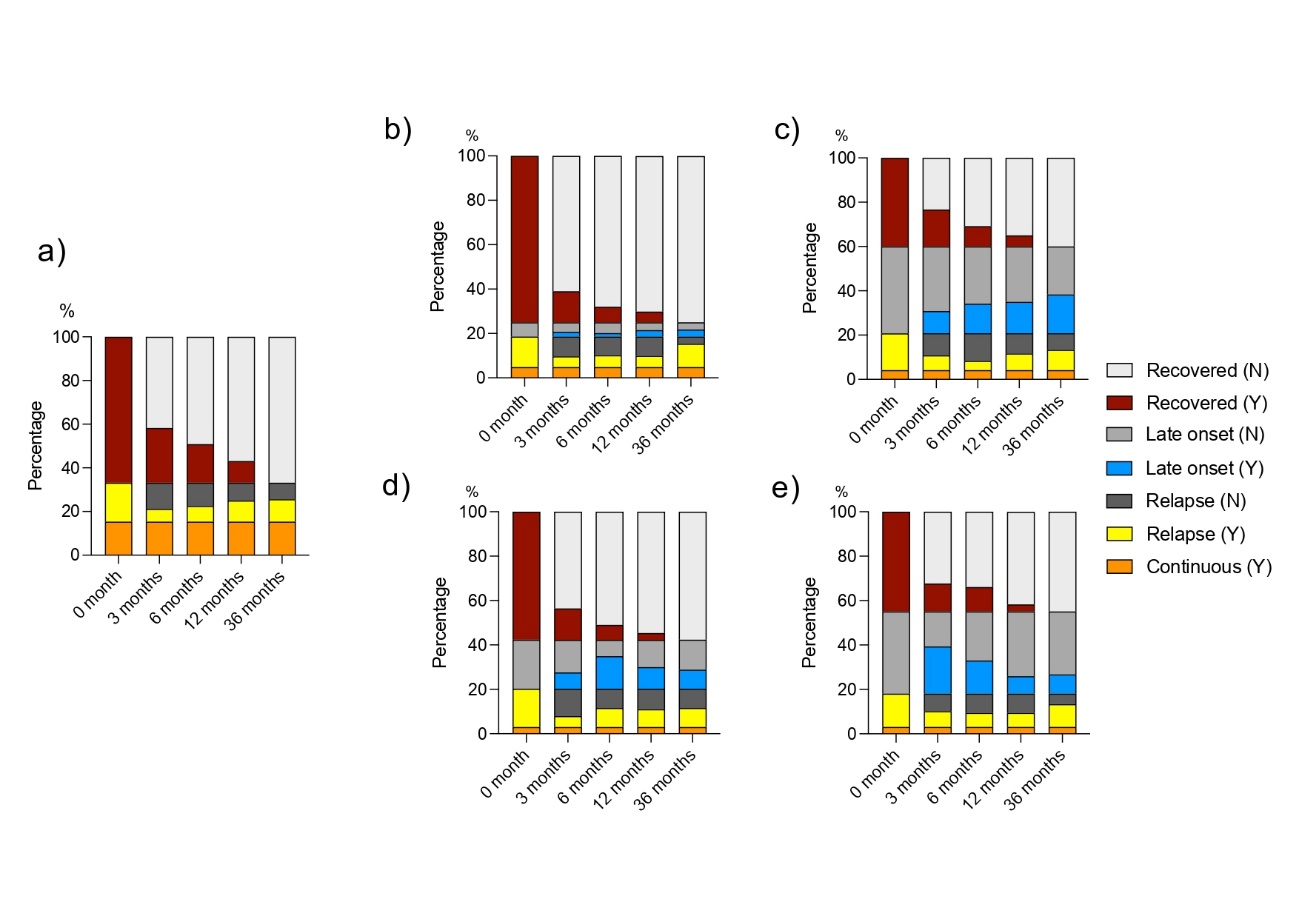


Percentage of a) at least one symptom, b) fatigue, c) memory impairment, d) concentration impairment, and e) alopecia at each time point. Y is the number of patients with symptoms, and N is that without symptoms.
